# Enhanced Magnetic Hyperthermia of Magnetoferritin through Synthesis at Elevated Temperature

**DOI:** 10.3390/ijms23074012

**Published:** 2022-04-04

**Authors:** Jiacheng Yu, Changqian Cao, Fengjiao Fang, Yongxin Pan

**Affiliations:** 1 Key Laboratory of Earth and Planetary Physics, Institute of Geology and Geophysics, Chinese Academy of Sciences, Beijing 100029, China; yujiacheng@mail.iggcas.ac.cn (J.Y.); fangfengjiao@mail.iggcas.ac.cn (F.F.); yxpan@mail.iggcas.ac.cn (Y.P.); 2 Innovation Academy for Earth Science, Chinese Academy of Sciences, Beijing 100029, China; 3 College of Earth and Planetary Sciences, University of Chinese Academy of Sciences, Beijing 100049, China

**Keywords:** hyperthermophilic archaeon, iron oxide nanoparticle, magnetic hyperthermia, magnetoferritin, biomineralization

## Abstract

Iron oxide nanoparticles have attracted a great deal of research interest in recent years for magnetic hyperthermia therapy owing to their biocompatibility and superior thermal conversion efficiency. Magnetoferritin is a type of biomimetic superparamagnetic iron oxide nanoparticle in a ferritin cage with good monodispersity, biocompatibility, and natural hydrophilicity. However, the magnetic hyperthermic efficiency of this kind of nanoparticle is limited by the small size of the mineral core as well as its low synthesis temperature. Here, we synthesized a novel magnetoferritin particle by using a recombinant ferritin from the hyperthermophilic archaeon *Pyrococcus furiosus* as a template with high iron atom loading of 9517 under a designated temperature of 90 °C. Compared with the magnetoferritins synthesized at 45 and 65 °C, the one synthesized at 90 °C displays a larger average magnetite and/or maghemite core size of 10.3 nm. This yields an increased saturation magnetization of up to 49.6 emu g^−1^ and an enhanced specific absorption rate (SAR) of 805.3 W g^−1^ in an alternating magnetic field of 485.7 kHz and 49 kA m^−1^. The maximum intrinsic loss power (ILP) value is 1.36 nHm^2^ kg^−1^. These results provide new insights into the biomimetic synthesis of magnetoferritins with enhanced hyperthermic efficiency and demonstrate the potential application of magnetoferritin in the magnetic hyperthermia of tumors.

## 1. Introduction

Magnetic nanoparticles (MNPs) have been applied widely in biomedical fields, including diagnosis and therapy, for their unique superparamagnetism and safety within the body [1]. In particular, MNPs are demonstrated to have an electromagnetic-thermal conversion capacity under an alternating magnetic field (AMF) via hysteresis or Néel/Brownian relaxation [2,3]. These properties ensure that MNPs have applications in magnetic hyperthermia therapy (MHT) for cancer treatment [4,5,6].

Ferritin is an iron-storage protein that is present in most living organisms and plays a significant role in iron management and detoxification for cellular iron homeostasis [7]. The structure of ferritin is nanocage-like with an outer diameter of 12 nm and an inner cavity diameter of 8 nm and is self-assembled by multiple polypeptide subunits (usually 24) [8,9]. In past decades, ferritin was used as an excellent biotemplate for the synthesis of magnetic cores (Fe_3_O_4_, γ-Fe_2_O_3_) in a ferritin cage to form a composite, called magnetoferritin. Magnetoferritin has been widely used in targeted drug delivery, magnetic resonance imaging contrast agents, and MHT for its excellent biocompatibility, monodispersity, and chemical/genetic modifiability [10,11,12,13]. However, its application is limited by the low magnetic-to-thermal conversion efficiency of conventional magnetoferritins. For example, Balejcikova et al. investigated the hyperthermic efficiency of magnetoferritins with a diameter of 4.2 nm under different AMF conditions (*f* = 190 kHz and *H* = 5.9–12.0 kA m^−1^) [14], but the magnetic heating effect was too low to calculate the specific absorption rate (SAR). That was explained by the small size and the low crystallinity of the polyphase core [14]. To improve the hyperthermic efficiency of magnetoferritin, we synthesized a human H chain magnetoferritin (MHFn) with a highly crystalline magnetite and/or maghemite core (4.8 nm) under strictly anaerobic conditions. The SAR value under the applied AMF (*f* = 805.5 kHz, *H* = 19.5 kA m^−1^) was 51.3 W g^−1^, and the intrinsic loss power (ILP) was 0.17 nHm^2^ kg^−1^ [15]. Fantechi et al. reported a cobalt doping method during the synthesis of MHFn to enhance the magnetic anisotropy and hyperthermic efficiency, but the SAR under the AMF (12.4 kA m^−1^, 183 kHz) was only 2.81 W g^−1^ [13]. For superparamagnetic nanoparticles with a diameter no greater than 20 nm, the heating efficiency remarkably increased with the particle size [16]. However, it is difficult to synthesize a magnetite core greater than 8 nm at a conventional synthesis temperature of 65 °C [17,18,19] because the internal diameter of the ferritin cage (~8 nm) limits the loading of iron atoms to <5000.

In this work, we synthesized magnetoferritin within a ferritin cage (PfFn) from the hyperthermophilic archaeon *Pyrococcus furiosus* by adding theoretical loading factors of 10,000 Fe/cage at temperatures of 45 °C, 65 °C, 90 °C, and 95 °C, named MPfFn-45, MPfFn-65, MPfFn-90, and MPfFn-95, respectively. PfFn is considered to be the most thermostable ferritin so far, with a melting temperature (T_m_) of >120 °C or 116.8 °C measured under different conditions [20,21]. Compared with mammalian ferritins, such as recombinant human H chain ferritin (T_m_ = 77 °C) [22], PfFn has a much higher thermostability as well as a different inner structure. In particular, the nucleation sites of PfFn contain fewer acidic amino acid residues, leading to the different magnetic behavior of obtained magnetoferritin [23,24]. Various characterization techniques, including transmission electron microscopy (TEM), dynamic light scattering (DLS), circular dichroism (CD) spectrum, and Fourier transform infrared (FTIR) spectra were used to determine the particle size, morphology, structure, and composition of magnetoferritin [25,26]. We found that the magnetoferritin can be obtained at a maximum temperature of 90 °C to achieve a magnetite core size of 10.3 nm, which exceeds the inner size of the ferritin cage (~8 nm). The core size, saturation magnetization, and hyperthermic efficiency of MPfFn are apparently improved as the synthesis temperature increases from 45 °C to 90 °C. The SAR and ILP values of MPfFn-90 are significantly higher than those of magnetoferritin and some MNPs with comparable sizes in previous works. This work provides new insights into the heating efficiency of magnetoferritin and the potential application in magnetic hyperthermia treatment of tumors and heat-triggered drug release.

## 2. Results and Discussion

### 2.1. Preparation and Characterization of MPfFn

**Figure 1:**
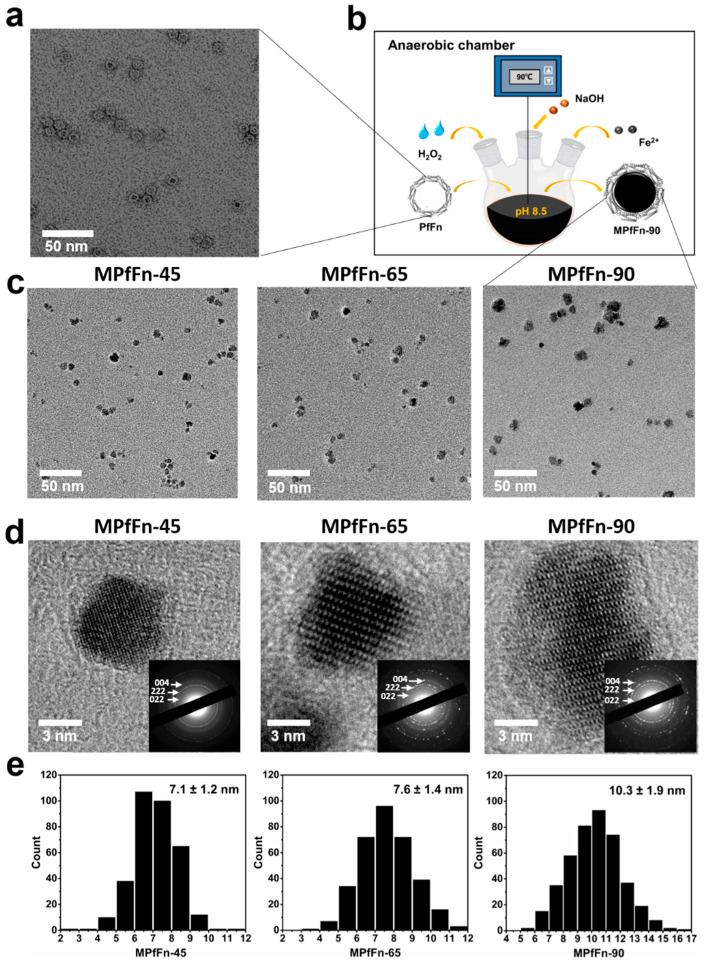
Structural characteristics of recombinant ferritin cages and MPfFn samples. (**a**) Negative-stained TEM images of PfFn (scale bar: 50 nm). (**b**) Schematic diagram of the biomimetic synthesis of MPfFn-90. (**c**) TEM images, (**d**) high-resolution TEM images, and (**e**) size distribution histograms of MPfFn-45, MPfFn-65, and MPfFn-90. The inset in Figure 1d is the selected area’s electron diffraction image, where the measured lattice planes (004), (222), and (022) indicate that the mineral structure is magnetite (Fe_3_O_4_) and/or maghemite (γ-Fe_2_O_3_).

The negative stained TEM image shows that PfFn is composed of a homogeneous spherical nanocage approximately 12 nm in diameter (Figure 1a). When theoretical 10,000 Fe^2+^ and H_2_O_2_ (mole ratio = 3:1) were simultaneously and stepwisely added into the PfFn solution under strictly controlled anaerobic conditions (Figure 1b), the synthesized MPfFn-45, MPfFn-65, and MPfFn-90 became a homogeneous black solution with very few precipitates, but the majority of the MPfFn-95 particles precipitated after centrifugation (Figure S1). As shown in the TEM images (Figure 1c), the iron oxide cores of the MPfFn-45, MPfFn-65, and MPfFn-90 were well dispersed with mean diameters of 7.1 ± 1.2 nm, 7.6 ± 1.4 nm, and 10.3 ± 1.9 nm, respectively (Figure 1e). The high-resolution TEM images and selected diffraction rings (Figure 1d) demonstrate that the inner cores of the three samples are highly crystalline magnetite (Fe_3_O_4_) and/or maghemite (γ-Fe_2_O_3_). It is worth noting that further characterizations to distinguish between the two minerals are not provided in this study. We think the magnetite/maghemite ratio of the magnetoferritin core has an extremely slight effect on the hyperthermic efficiency because of the similarity in the crystal structure and magnetic properties of the two minerals [27]. As seen in Figure S2, severe aggregation of MPfFn-95 occurred when the temperature was elevated to 95 °C. Based on the yield of monodispersed magnetoferritin particles, we consider 90 °C an appropriate temperature for synthesis of MPfFn particles in our method, and this synthesis temperature is much higher than that in most previous works [17,19,23,28]. This can be explained by the extreme living conditions of *Pyrococcus furiosus*, which grows between 70 °C and 103 °C and at pH values between 5 and 9, with optimal growth conditions of 100 °C and pH 7, corresponding to the shortest doubling time of 37 min [29]. In our previous study, the ferritin cage (PfFn) was demonstrated to maintain its iron incorporation function after being heated at the temperature of 110 °C for 30 min [20]. However, the large iron loading factors (10,000/protein cage) and the long reaction time (200 min) can change the structure of the ferritin and partially weaken its shell stability [20,30] resulting in the good dispersion of the MPfFn-90 but aggregation of the MPfFn-95.

To quantify the real loading factors of iron in the ferritin cage of the MPfFn-45, MPfFn-65, and MPfFn-90, the ferritin cage and iron contents were determined by thermogravimetric analysis (TGA) and the Ferrozine method [31], respectively. The ferritin cage and Fe percentages of these particles are listed in Table S1 (see Supplementary Materials). Accordingly, we calculated that the ferritin cages of MPfFn-45, MPfFn-65, and MPfFn-90 contain 5494, 6737, and 9517 Fe atoms, respectively.

**Figure 2:**
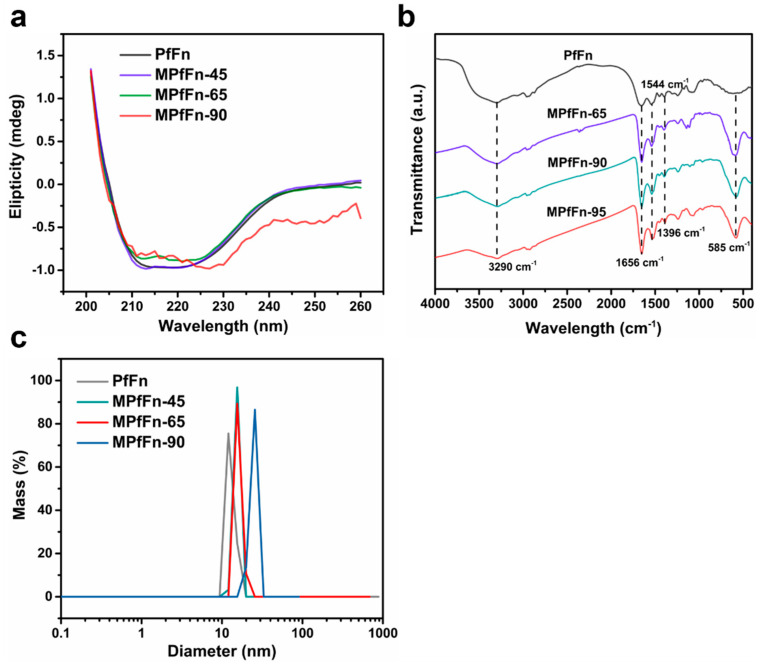
(**a**) CD spectra of the PfFn and MPfFn samples. (**b**) FTIR spectra of PfFn and MPfFn samples. (**c**) DLS analysis of PfFn, MPfFn-45, MPfFn-65, and MPfFn-90 samples with hydrodynamic diameters of 13.8, 15.4, 16.0, and 25.0 nm, respectively.

As the average core size of MPfFn-90 exceeds the inner diameter of the PfFn cage (~8 nm), it is crucial to ascertain whether the protein cage is perturbed for the reason that the functional groups (i.e., -NH_2_, -COO-, -OH) on the protein cage can be used for chemical modification for diagnostics and therapeutics [32,33]. The CD spectra revealed that the secondary structure of MPfFn-45 was well maintained (Figure 2a), whereas that of MPfFn-65 changed slightly and that of MPfFn-90 changed significantly. This indicates that long-term mineralization (200 min) at a high temperature influences the outer protein structure of magnetoferritins [34]. However, the FTIR spectra (Figure 2b) show that the absorption peaks appearing at 1654 cm^−1^ and 1544 cm^−1^ in the PfFn and MPfFn samples are specific signals of the peptide bond and correspond to amides 1 and 2, respectively [35,36]. The characteristic peak (1396 cm^−1^) of -COO stretching vibrations of amino acid side chains [37] and the board characteristic band (~3290 cm^−1^) of -NH_2_ and -OH stretching vibrations [38] were retained after biomineralization. These results demonstrate that the functional groups are maintained well in all samples. Moreover, the DLS data show that the hydrodynamic diameter (HD) of apoferritin PfFn (13.8 nm) slightly increases to 15.4, 16.0, and 25.0 nm after biomineralization of MPfFn-45, MPfFn-65, and MPfFn-90, respectively (Figure 2c). This demonstrates that all magnetoferritin particles are monodispersed due to the outer ferritin cage. Accordingly, we speculated that the high thermostability and flexibility allows the PfFn cage to remain intact while becoming somewhat larger during mineralization at 90 °C, resulting in a larger core size.

### 2.2. Magnetic Hyperthermia of Magnetoferritin

**Figure 3:**
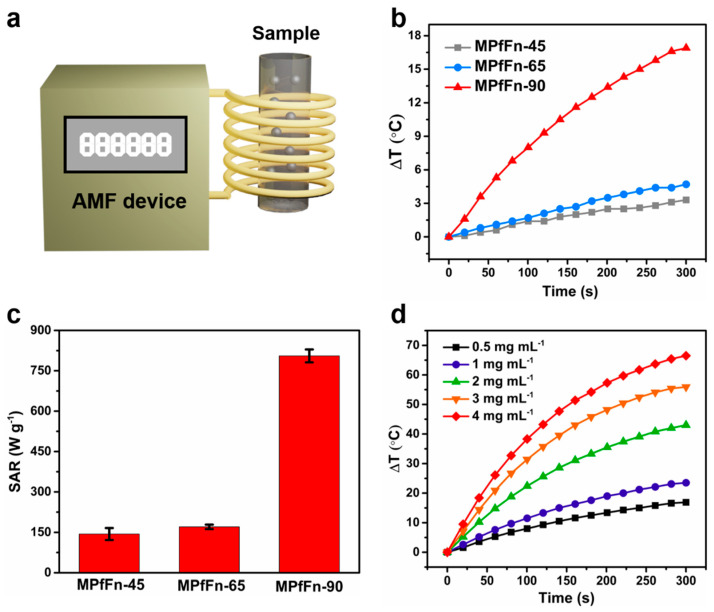
Hyperthermic performance of MPfFn under AMF. (**a**) Schematic illustration of the magnetic hyperthermia test. (**b**) Time-dependent temperature change curves of MPfFn-45, MPfFn-65, and MPfFn-90 under an AMF (= 485.7 kHz, *H* = 49 kA m^−1^). (**c**) Comparison of SAR values. (**d**) Time-dependent temperature change curves with different iron concentrations.

To investigate the magnetically induced heating capability of the MPfFn samples, we measured the hyperthermia performance by recording the temperature kinetics of their colloidal dispersions under exposure to a designated AMF (*f* = 485.7 kHz, *H* = 49 kA m^−1^) (Figure 3a). Samples were kept at the same Fe concentration (0.5 mg mL^−1^) for direct comparison. The initial sample temperature of the experiment was kept at about 22 °C to minimize the influence of the coil temperature on the sample. MPfFn-90 demonstrated an excellent hyperthermia effect, with a temperature rise of about 16.8 °C for 5 min (Figure 3b). In contrast, the temperatures of MPfFn-45 and MPfFn-65 only increased by approximately 3.3 and 4.7 °C, respectively. As shown in Figure 3c, the SAR value of MPfFn-90 was as high as 805.3 W g^−1^, 5.6 times higher than that of MPfFn-65 (143.6 W g^−1^) and 4.7 times higher than that of MPfFn-65 (170.4 W g^−1^). Furthermore, the iron concentration-dependent heating effect of MPfFn-90 was also investigated. As shown in Figure 3d, it was found that ΔT of MPfFn-90 significantly increased with the increase in iron concentration; it reached 16.9 °C, 23.5 °C, 43 °C, 55.9 °C, and 66.5 °C in 300 s for concentrations of 0.5, 1, 2, 3, and 4 mg mL^−1^, respectively.

**Figure 4:**
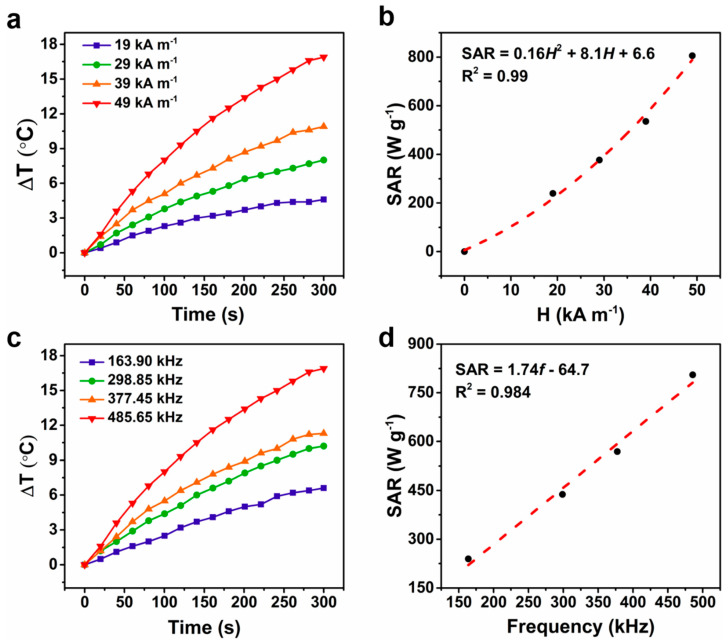
Effects of magnetic field intensities (*H*) and AC frequencies (*f*) on the hyperthermia performance of MPfFn-90. Time-dependent temperature change curves with varying (**a**) *H* and (**c**) *f*. SAR values as a function of (**b**) *H* and (**d**) *f*.

The ΔT under an AMF with *f* = 485.65 kHz and varying intensity is shown in Figure 4a. As expected, higher SAR values were recorded by increasing the applied *H* and followed a square trend (Figure 4b). Further, ΔT under *H* = 49 kA m^−1^ with varying *f* is shown in Figure 4c. A linear trend was also observed whenever the SAR values were plotted as a function of the applied *f* at a fixed *H* (Figure 4d). The behavior of SAR versus *H* and *f* is in agreement with that observed in previous studies [39,40]. To compare the heating efficiency of our samples with that of MNPs reported in previous studies, the ILP (normalized SAR) was calculated according to Equation (1).

ILP = SAR/(*fH*^2^) (1)

Table 1 compares the ILP-related parameters of our results with other works. It demonstrates that the maximum ILP value of MPfFn-90 (1.36 nH m^2^ kg^−1^) is not only much higher than that of reported magnetoferritins [13,15,24], but also higher than some reported MNPs with comparable core sizes, indicating that MPfFn-90 has better hyperthermic efficiency and thus shows great potential in magnetic hyperthermia applications.

### 2.3. Magnetic Properties of Magnetoferritin Nanoparticles

**Figure 5:**
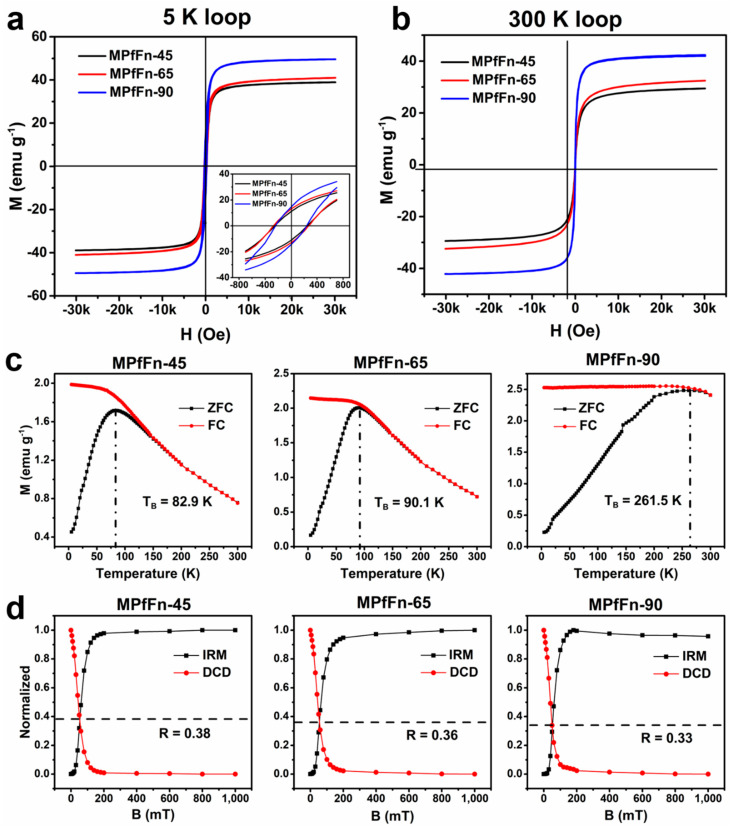
Magnetic characterization of MPfFn nanoparticles. Hysteresis loops of the MPfFn samples measured at (**a**) 5 K and (**b**) 300 K. (**c**) ZFC/FC magnetization curves of the three samples. (**d**) Normalized IRM acquisition and DCD curves of the three samples.

To better understand the hyperthermic mechanisms of MPfFn samples, the magnetic properties of the samples were investigated, and the results are shown in Figure 5 and Table 2. The hysteresis loops measured at 300 K display zero coercivity (*H_c_*), confirming that all samples are typical superparamagnetic nanoparticles. The saturation magnetization (*M_s_*) of MPfFn-90 (42.3 emu g^−1^) is much higher than that of MPfFn-45 (29.4 emu g^−1^) and MPfFn-65 (32.4 emu g^−1^). Conversely, at a low temperature (5 K) all samples display open hysteretic loops. The *H_c_* values of MPfFn samples are in accordance with that of magnetite or maghemite within 200–300 Oe [47]. The MPfFn-90 also shows the highest *M_s_* value among these samples at 5 K. A high value of *M_s_* can make MNPs convert more electromagnetic energy into heat energy under an applied AMF [43], which can partially explain the higher SAR value of MPfFn-90. The zero-field-cooled (ZFC) and field-cooled (FC) magnetization curves in a field of 1.5 mT from 5 K to 300 K are shown in Figure 5c. The blocking temperature (T_B_) of MPfFn-90 (261.5 K) is much higher than that of MPfFn-45 (82.9 K) and MPfFn-65 (90.1 K), indicating that MPfFn-90 hasa larger core size. This is in good agreement with the TEM results. Based on the Wohlfarth–Cisowski test for randomly oriented noninteracting single-domain particles, the isothermal remanent magnetization (IRM) acquisition curve and direct current demagnetization (DCD) curve intersect at R = 0.5 [48]. In this work, the R values (Figure 5d) of MPfFn-45, MPfFn-65, and MPfFn-90 are 0.38, 0.36, and 0.33, respectively. This decrease in R value suggests more magnetostatic interactions with the larger core of MPfFn-90. The magnetic properties of MPfFn-95 were also studied, and the results are shown in Figure S3. Compared with MPfFn-90, MPfFn-95 exhibits a higher *M_s_* value at both 5 K and 300 K, which might be due to the higher synthesis temperature. However, no T_B_ value is observed, indicating a larger core caused by particle aggregation.

**Figure 6:**
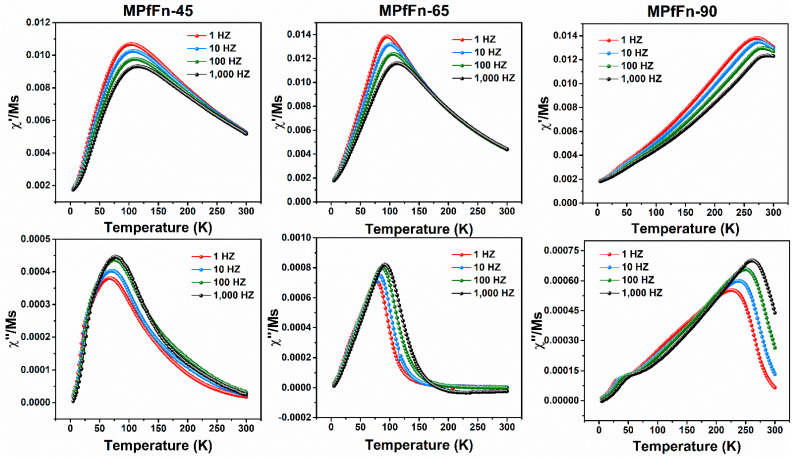
AC susceptibility of MPfFn samples performed with various logarithmic spaced frequencies in the 1–1000 Hz range. The upper and lower figures are the in-phase (χ′) and out-of-phase (χ″) susceptibility, respectively.

**Figure 7:**
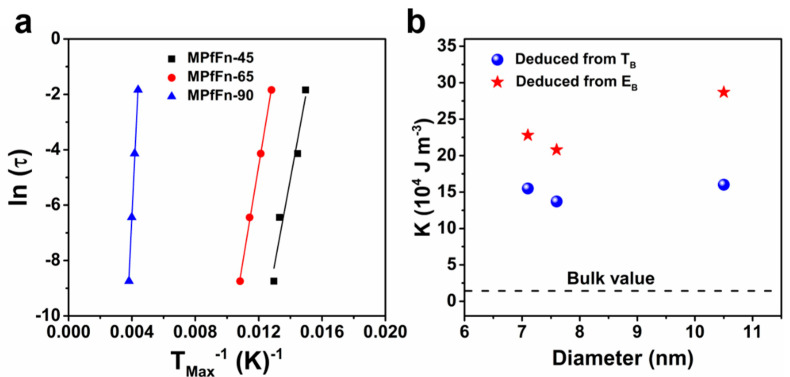
(**a**) Linear plot of the inverse of maximum temperature (1/T_Max_) of each frequency according to the Arrhenius law. (**b**) Effective magnetic anisotropy values (K_eff_) of MPfFn-45 (7.1 nm), MPfFn-65 (7.6 nm), and MPfFn-90 (10.5 nm) obtained from E_B_ (red stars) and T_B_ (blue dots). The black dotted line represents the bulk value of E_B_.

Magnetic anisotropy is also a significant parameter that influences the hyperthermic efficiency of MNPs [49]. With the determined T_B_ value, the anisotropy constant K_A_ of MNPs with no magnetic interactions can be calculated by [50].

(2) $$K_{A\;}=\;\ln(\frac{t_{0}}{\tau_{0}})\frac{k_{B}T_{B}}{V}$$

where t_0_ is the timescale of the measurement (100 s), τ_0_ is the microscopic jump time (10^−9^ s), k_B_ is the Boltzmann constant, and V is the volume of MNPs, which can be estimated using the average particle diameter determined via TEM measurements (spherical shape approximation). The K_A_ values obtained for MPfFn-45, MPfFn-65, and MPfFn-90 are 1.55 × 10^5^, 1.37 × 10^5^, and 1.60 × 10^5^ J m ^−3^, respectively. However, we noticed that the magnetic interactions among the MPfFn samples are nonnegligible, and such interactions influence the real value of T_B_ [51]. Therefore, the AC susceptibility at temperatures from 5 K to 300 K under an AC field of 1 to 1000 Hz at 4 Oe was studied. The in-phase (χ′) and out-of-phase (χ″) susceptibility of the three samples at different excitation frequencies are displayed in Figure 6 According to the Néel model, the temperature dependence of the relaxation of the magnetization of noninteracting superparamagnetic systems follows an Arrhenius law [52].

(3) $$\tau \left(T\right)=\tau_{0}\exp\left(E_{B}/k_{B}T\right)\;$$

where E_B_ is the anisotropy energy barrier for magnetization reversal, E_B_ = K_eff_ V, and K_eff_ is the effective anisotropy constant. τ_0_ is the attempt time. The plot of ln (τ) = ln (1/2πν) versus of 1/T_Max_ (the blocking temperatures obtained from the χ″ maxima for different observation times) is a straight line (Figure 7a), consistent with an Arrhenius law. The E_B_ and τ_0_ values are listed in Table 2, and the effective anisotropy values K_eff_ deduced from E_B_ are approximately 2.28 × 10^5^ J m ^−3^, 2.08 × 10^5^ J m ^−3^, and 2.87 × 10^5^ J m ^−3^, respectively (Figure 7b). It is clear that the values of K_eff_ for all MPfFn samples are higher than that for bulk magnetite (1.35 × 10^4^ J m ^−3^) [16,51]. It is worth noting that the anisotropy constant includes the contributions from crystalline, shape, and surface anisotropy [17]. The magnetic nanoparticles (6–11 nm) showed decreasing K_eff_ with size in previous work [17,51], and the variation is mainly attributable to changes in their size. In contrast, the K_eff_ in our work decreased when the size of MPfFn increased from 7.1 to 7.6 nm but increased drastically when the size increased from 7.6 to 10.3 nm. The shape anisotropy can be ignored because of the similar shape of magnetoferritins. Besides the surface anisotropy affected by the size, crystalline anisotropy is also an important factor that influences the K_eff_ of magnetoferritins. The elevated temperature of 90 °C largely increased the crystalline anisotropy, resulting in a higher K_eff_.

## 3. Materials and Methods

### 3.1. Materials

Ammonium ferrous sulfate ((NH_4_)_2_Fe(SO_4_)_2_·6H_2_O) was purchased from Aladdin (Shanghai, China). Sodium chloride, Tris, and sodium hydroxide were obtained from Sangon Biotech (Shanghai, China). All the water used in experiments was supplied by a Milli-Q system (Merck KGaA, Darmstadt, Germany). 3.2. Expression and Purification of PfFn

Recombinant PfFn was prepared as previously described [20]. Briefly, the expression vector pET-22b containing the *PfFn* gene was transformed into *Escherichia coli* BL21 (DE3). The *E. coli* cells were cultured at 37 °C to an OD600 of 0.6 in ampicillin-containing liquid Luria–Bertani (LB) medium and induced expression with 0.5 mM isopropyl-b-d-thiogalactoside (IPTG) overnight at 30 °C. The cells were harvested by centrifugation at 8000 rpm for 8 min, and the pellet was washed once and resuspended in Tris–HCl buffer (0.025 M Tris, 0.1 M NaCl, pH 8.5). The cells were then incubated in lysis buffer (1 mM EDTA, 50 mg mL^−1^ lysozyme, 0.025 M Tris, 0.1 M NaCl, pH 8.5) for 2 h at 37 °C. The purification process of PfFn was conducted by heating the lysate at 100 °C for 25 min with constant stirring. The purified proteins were obtained by collecting the supernatant after centrifugation at 20,000 g for 40 min. Finally, the purified PfFn was desalted with 0.1 M NaCl buffer for further synthesis of MPfFn particles. Protein concentrations were determined by a Pierce™ BCA protein assay kit.

### 3.2. Synthesis of MPfFn under Different Temperatures

Four batches of prepared solution of purified PfFn (100 mL, 1 mg mL^−1^) in 0.1 M NaCl in reaction vessels were degassed and transferred to an anaerobic chamber. A total of 50 mM (NH_4_)_2_Fe(SO_4_)_2_·6H_2_O as iron source and 16.67 mM H_2_O_2_ as oxidant were dissolved in degassed water (200 mL) respectively. The Fe_3_O_4_ formation reaction can be expressed as,

3Fe^2+^ + H_2_O_2_ + 2H_2_O →Fe_3_O_4_ + 6H^+^ (4)

For the synthesis of MPfFn-45 with theoretical loading factors of 10,000 Fe/cage, 41.46 mL of (NH_4_)_2_Fe(SO_4_)_2_·6H_2_O solution and 41.46 mL of H_2_O_2_ solution were simultaneously added into the prepared PfFn solution at a rate of 50 Fe/(protein min) using a dosing device (800 Dosino). The reaction temperature was maintained at 45 °C, and the pH was stabilized at 8.5 by 100 mM NaOH solution with a pH stat titrator. After 200 min of titration, the reaction was finished. 1 mL of 0.3 M sodium citrate was added to chelate any free iron species. Finally, the MPfFn-45 solution was obtained after centrifugation (10,000× *g*) for 10 min. Similarly, MPfFfn-65, MPfFn-90, and MPfFn-95 were synthesized with the same procedure under 65 °C, 90 °C, and 95 °C, respectively.

### 3.3. Characterization of MPfFn Particles

The morphology and crystallography of MPfFn were analyzed by TEM (JEOL JEM-2100, Tokyo, Japan) with an accelerating voltage of 200 kV. The size distribution of the magnetoferritins was measured over 300 particles, and crystallographic orientation of the core was examined by high-resolution TEM (HR-TEM). For negative staining TEM observation, apoferritin and magnetoferritin samples (3 μL, 0.2 mg ml^−1^) were embedded in a Plasma Cleaner HPDC32G treated copper grid and stained with 1% uranyl acetate for 1 min then imaged with a JEM-1400 100-kV TEM (JEOL, Tokyo, Japan). The hydrodynamic sizes of PfFn and MPfFn were determined by DLS (DynaPro NanoStar, Wyatt Technology Corporation, Santa Barbara, CA, USA) at 25 °C with a scattering angle of 90°. TGA was used to obtain the proportion of ferritin cage of entire magnetoferritin nanoparticles by using a thermogravimetric analyzer (TGA/DSC 3 STARe Mettler Toledo). Samples were heated from 30 °C to 800 °C at 5 °C min^−1^ under N_2_ flow at 50 mL min^−1^. N_2_ flow is used to prevent the further oxidation of Fe_3_O_4_. The iron concentration of the solutions was determined by a ferrozine method [28]. FTIR spectroscopy (Thermo Fisher Nicolet 6700 spectrometer) characterization was performed using potassium bromide, and the FTIR spectra of the prepared samples were recorded in the range from 4000 to 400 cm^−1^.

### 3.4. Magnetic Measurements of MPfFn

The desalted MPfFn nanoparticles were freeze dried, and magnetic measurements of the dried samples were conducted with a magnetic property measurement system (MPMS-5XL, Quantum Design Inc., San Diego, CA, USA). The IRM acquisition and DCD curves were measured at 5 K within 0–1 T to calculate the magnetostatic interactions. The ZFC and FC curves were measured in a 1.5 mT field from 5 K to 300 K, and the blocking temperature (T_B_) was determined from the maximum of the ZFC curves. Hysteresis loops were measured in the field range of ±3 T at 5 K and 300 K. The AC susceptibility was measured at frequencies of 1, 10, 100, and 1000 Hz within a temperature range of 5 K to 300 K in a weak field of 0.4 mT.

### 3.5. Hyperthermic Efficiency Analyses

The hyperthermic efficiency of MPfFn samples was measured with a commercial system D5 series device (nB nanoScale Biomagnetics, Zaragoza, Spain). Each MPfFn aqueous sample (1 mL) with a Fe concentration of 0.5 mg mL^−1^ was placed in the middle of coil in a 2 mL glass chromatography vial. The temperatures of all samples during magnetic treatment were recorded by an optic-fiber temperature probe. The initial temperature of each sample was controlled and stabilized to 22 °C. The hyperthermic efficiency, expressed in terms of SAR, was calculated.

(5) $$\mathrm{SAR}=\frac{\mathrm{CV}}{m\left(\mathrm{Fe}\right)}\frac{\mathrm{dT}}{\mathrm{dt}},$$

where C is the volumetric specific heat capacity of water (4.185 J g^−1^ K^−1^), V is the sample volume (1 mL), and m (Fe) is the total mass of Fe in the sample. dT/dt is the initial slope of the ΔT curve as a function of time, which was determined by fitting the curves of field application time vs. temperature with the Box–Lucas equation, T(t) = A(1−e^−Bt^) [15]. The ILP was calculated using ILP = SAR/(*f·H*^2^).

## 4. Conclusions

Four kinds of magnetoferritins were fabricated by using PfFn as a template under different temperatures (45 °C, 65 °C, 90 °C, and 95 °C), named MPfFn-45, MPfFn-65, MPfFn-90, and MPfFn-95, respectively. The TEM images show that 90 °C is the highest temperature for synthesis of monodispersed magnetoferritin particles through our method. The core size, magnetic properties, and magnetic hyperthermic efficiency of magnetoferritin show an increasing trend with the synthesis temperature increase. The MPfFn-90 shows a highest SAR value of 805.3 W g^−1^ with a maximum ILP value of 1.36 nH m^2^ kg^−1^; the values are significantly higher than those of other magnetoferritins and some MNPs with the same size range. The MPfFn-90 with enhanced hyperthermic efficiency might show good application prospects in magnetic hyperthermia treatment and heat-triggered drug release.

## Figures and Tables

**Table 1:** Comparison of TEM size, magnetic field intensity, frequency, and SAR values of the MPfFn with other MNPs.

Sample	TEM Size (nm)	*H* (kA m^−1^)	*f* (kHz)	SAR (W g^−1^)	ILP (nHm^2^ kg^−1^)	Reference
HFt5	6.8	12.4	183	2.81	0.1	[13]
PfFt10		12.4	183	4.9	0.17	[24]
MHFn	4.8	19.5	805.5	51.3	0.17	[15]
AFF-3	10	13.9	175.2	48.6	1.4	[41]
MMNPs	10	40.56	300	127.7	0.26	[42]
Fe_3_O_4_	6.5	4	165.3	10.3	3.8	[43]
MNPs	13	13.8	114	14.1	0.65	[44]
Pro-Glu-MNPs	4.5	42.3	300	69	0.2	[45]
S_4_-Zn_0.53_ Fe_2.47_O_4_@PEG	20	24	765	380	0.86	[46]
MPfFn-45	7.1	49	485.7	143.6	0.12	This work
MPfFn-65	7.6	49	485.7	170.4	0.15	This work
MPfFn-90	10.3	49	485.7	805.3	0.70	This work
MPfFn-90	10.3	39	485.7	535.4	0.72	This work
MPfFn-90	10.3	29	485.7	376.8	0.92	This work
MPfFn-90	10.3	19	485.7	239.2	1.36	This work

**Table 2:** Comparison of magnetic parameters for MPfFn-45, MPfFn-65, and MPfFn-90.

								Arrhenius Law	
Samples	Core Size (nm)	HD (nm)	*M_s_* (300 K) (emu g^−1^)	*M_s_* (5 K) (emu g^−1^)	*H_c_* (5 K) (Oe)	T_B_ (K)	R	E_B_/k_B_ (K)	τ0 (s)
MPfFn-45	7.1	15.4	29.4	38.7	256.9	82.9	0.38	3102.5	8.6 × 10^−22^
MPfFn-65	7.6	16.0	32.4	41.0	273.7	90.1	0.36	3461.0	9.5 × 10^−21^
MPfFn-90	10.3	25.0	42.3	49.6	240.1	261.5	0.33	11,913.0	3.2 × 10^−24^

## Data Availability

Additional data related to this paper are available upon request to the authors.

## Supplementary Materials

The following supporting information can be downloaded at: https://www.mdpi.com/article/10.3390/ijms23074012/s1.
