# Supplementary material for: Enhanced Magnetic Hyperthermia of Magnetoferritin through Synthesis at Elevated Temperature
Source: Int J Mol Sci. 2022 Apr 4;23(7):4012. doi: 10.3390/ijms23074012 (PMC8999155; doi:10.3390/ijms23074012)
Supplement: Supplementary file 1 [file ijms-23-04012-s001.zip › ijms-1673380-supplementary.pdf]

Supplementary Materials

Enhanced Magnetic Hyperthermia of Magnetoferritin through Synthesis at Elevated Temperature

Jiacheng Yu [1,2], Changqian Cao [1,2,*], Fengjiao Fang [1,2,3] and Yongxin Pan [1,2,3]

[1] Key Laboratory of Earth and Planetary Physics, Institute of Geology and Geophysics, Chinese Academy of Sciences, Beijing 100029, China; yujiacheng@mail.iggcas.ac.cn (J.Y.); fangfengjiao@mail.iggcas.ac.cn (F.F.); yxpan@mail.iggcas.ac.cn (Y.P.)

[2] Innovation Academy for Earth Science, Chinese Academy of Sciences, Beijing 100029, China

[3] College of Earth and Planetary Sciences, University of Chinese Academy of Sciences, Beijing 100049, China

* Correspondence: changqiancao@mail.iggcas.ac.cn

Table S1. Ferritin cage and iron content of MPfFn samples.

Sample	PfFn cage (wt.%)	Fe (wt.%)	[a] Number (Fe atom/ferritin cage)
MPfFn-45	46.4	29.3	5494
MPfFn-65	35.9	27.8	6737
MPfFn-90	29.8	32.6	9517

[a] Number = (Fe %/56)/(PfFn cage %/MW_{PfFn}), $MW_{PfFn\ cage}$ = 20.3KD*24mer=487200 g mol^{-1}.

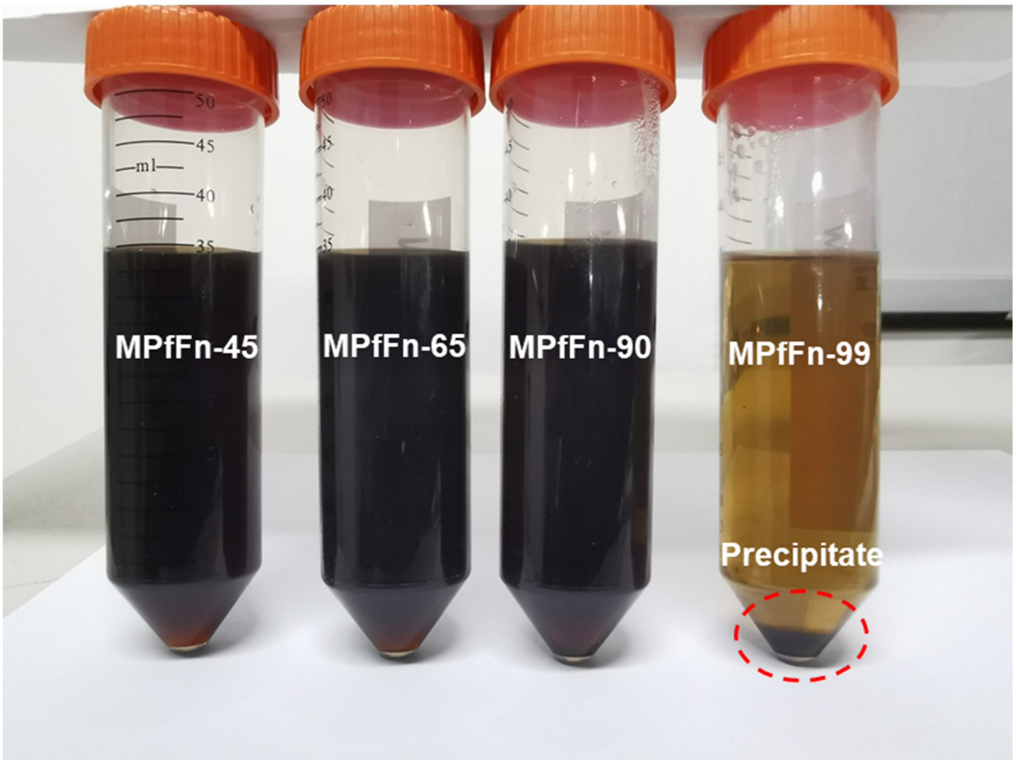

Figure S1. The images of MPfFn solution after synthesis at different temperature.

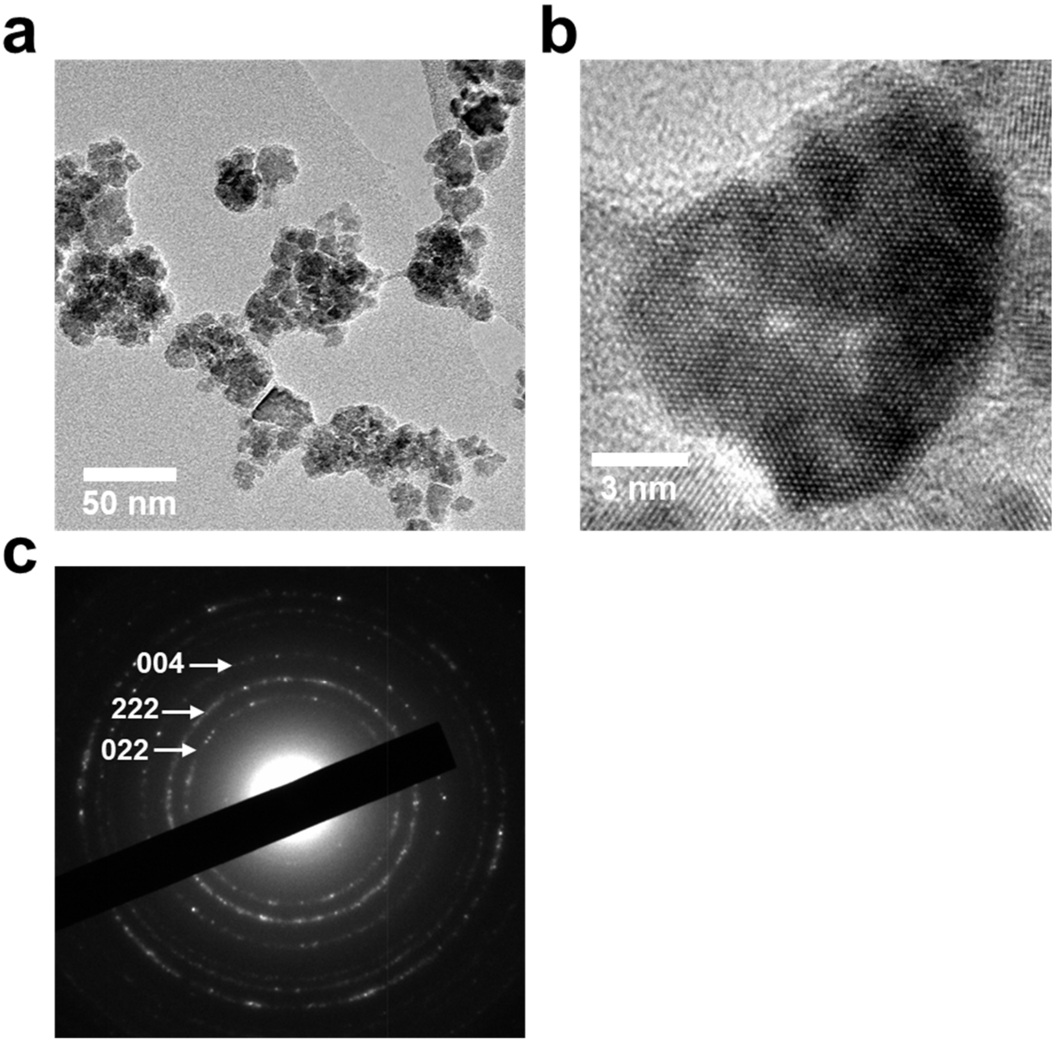

Figure S2. Structural characteristics of MPfFn-95. (**a**) The TEM image, (**b**) High-resolution TEM image, (**c**) The selected area electron diffraction image, the measured lattice planes (004), (222), and (022).

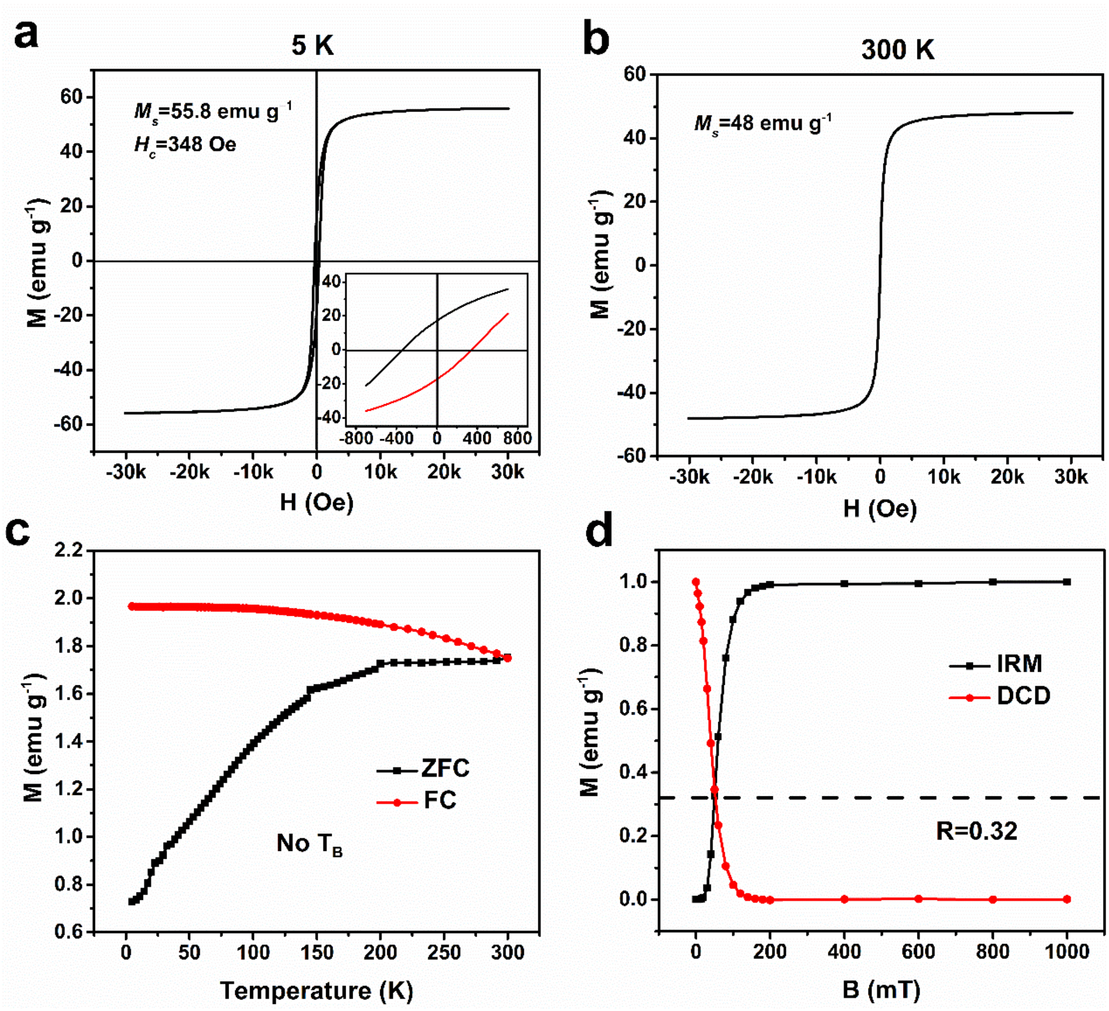

Figure S3. Magnetic characterization of MPfFn-95. Hysteresis loops measured at 5 K (**a**) and 300 K (**b**). (**c**) ZFC/FC magnetization curves. (**d**) Normalized IRM acquisition and DC demagnetization (DCD) curves of the MPfFn-95.
